# Topics of Public Interest

**Published:** 1913-09

**Authors:** 


### TOPICS OF PUBLIC INTEREST.

Prolongation of Life. The medical profession is often accused of a lack of practical accomplishment. The following statistics, compiled by Dr. John S. Billings from Census statistics, show results whose genuineness can scarcely be questioned and for which the medical profession may rightly claim most of the credit. Comparing the mortality tables of New York City for

1879-81 with those for 1909-1911, to insure fair averages, the average expectation of life is found to have changed as follows in thirty years:

| | 1879-81 | 1909-1911 |
|---|---|---|
| At 5 years of age or under........ | 41.0 years | 52.0 years |
| Between 5 and 10 years.......... | 46.0 years | 51.0 years |
| Between 25 and 30 years.......... | 32.0 years | 34.3 years |

Unfortunately, at later years, the average expectancy shows a slight diminution, ascribed to the prevalence of renal disease, arterio-sclerosis and cancer, all of which are due more or less to the increased strain of life and, in our opinion, to the fact that 30 years ago the persons who passed the age of 30 and subsequent higher ages, included a disproportionate number of those who had survived the various diseases, now prevented or cured, by virtue of inherent vitality and strength.

---

ANESTHESIA AMONG THE INDIANS. The anterior history on ancient anesthetic agents and methods is, with some difference, similar to the works of other authors and writers on this topic; but the following research of the "Anesthesia Among the Indians," is original of Dr. J. M. G. Kukay, as will be shown in this course.

It is very difficult to write on any subject of remote times without a manuscript or some other information to give some light on the writer's investigation, excepting mummies, trephined skulls and the revelation of some far descendants of the Incas Indians.

It is not possible to know the date and name of the first that discovered anesthesia in civilized Europe; Is it possible to know this in the savage region of America prior to Columbian time? The following brief history is the way taken by the writer in his research:

In the year 1904, I have a few pages of this work, and anything in regard to this subject was a point of investigation and study for me. When visiting our "American Museum of Natural History" in New York, my attention was called to the collection of Indian Mummies, buried with a sack of coca leaves, and the trephined skulls, brought by Mr. E. G. Squier from the burials of the Incas Indians at Chiara Valley, Peru.

My first step in this research was a visit to Peru, which I did one year after. Once in the Valley of Yucay, I began to ask the natives if they knew the use of coca among the Indians, but no one knew anything about it. Finally I got the address of an Indian's descendant, by name Senor Juan, who lived several miles away, and I went to his camp, accompanied by a native.

Senor Juan was about 90 years of age (he don't know it). After I told him the object of my visit (the writer is familiar with the Spanish language), he says: "We don't use books or any writing, but we keep all the records in the "Quipu" in which, by the color of the cord, length, number of knots, and kind of it, etc., we record the important incidents, and by memory, the non-important ones." "Can you tell me what for your forefathers were buried with coca leaves?" I asked. "Certainly," he answered. "Before the Spaniards came we were more happy and rich, and when a 'Cacique' (Indian Chief) or some member of his family dies, the relatives put with many other things the holly leaves." "What other use have they for these leaves?" "Oh, many," he said. "We use it even today as a tonic, to relieve pain, and chew it as you do tobacco." "Can you explain the reason of the perforation of the skull?" "Of course," he answered. This was done to the son or son-in-law of the dead 'Cacique' and was a religious ceremony. If the son lived after the operation he became cacique, but the most died after cutting the square hole in the head." "Do they use any coca leaves in the operation?" I asked. "Yes; plenty. The 'Curanderos' (Indian physicians) mixed in one concave stone a large quantity of leaves and water, and with the other stone, like a pestle, triturated them very fine to the consistence of syrup. Then they gave slowly to drink until they do not move, and the curanderos start the operation, which takes sometimes a whole day; and all the time during the operation the men, women and children pray with their prayer sticks."

"What do they do in case that the man feels pain?" "Well, they give him more stuff to drink." "Did they die before the operation was over on account of the syrup?" "Very few. The curanderos have very much experience in handling the coca, and they know the danger by the color of the skin."

"Have you seen any of such operations?" I asked. "No; they were used when the Incas domained this country, or in times when no christian inhabited here." "How do you know all their customs among your people?" "Easy. Some by memory from my fathers, grandfathers, etc., and others by old 'Quipus.'"

After all this caudal of information the writer went to Surco, Peru. In this place he compiled more information on the use of coca leaves; between the different uses was that they gave coca leaves to the Indian women to eat in labor time. But nobody gave so complete and important information as Senor Juan.

By this revelation, and the evidence from the museum, the writer was completely convinced that coca leaves were used to produce Narcosis and Anesthesia by the Incas Indians of Peru in performing surgical operations, by means of knives made from copper, bronze and stone.

No one who knows the works of Dr. von Tschudi, Mr. E. G. Squier, and the writer's research, will doubt this statement.

---

Department of Agriculture Advises That Milk be Pasteurized at Low Temperatures. In order to determine the best way of pasteurizing milk so as to kill the disease germs and yet not give the milk a cooked flavor or lessen its nutritive value, the Department of Agriculture, through its Dairy Division, has been conducting a series of experiments, treating milk at different temperatures and for different lengths of time. According to the report on these experiments in Bulletin 166 of the Bureau of Animal Industry, when milk is pasteurized at 145°F. for thirty minutes the chemical changes are so slight that it is unlikely that the protein or the phosphates of lime and magnesia are materially changed.

Moreover, from a bacteriologic standpoint, pasteurizing at low temperatures is found to be more satisfactory than at high temperatures. When low temperatures are used the majority of bacteria that survive are lactic acid organisms which play an important part in the normal souring of milk. When milk is efficiently pasteurized at high temperatures, the bacteria which survive are largely of the putrefactive kinds, and milk so treated if kept for any length of time has a tendency to rot instead of sour. From the standpoint of economy, the technologist of the Dairy Division finds that pasteurizing at 145°F. calls for about 23½ per cent. less heat. A similar saving of ice occurs.

The Department, therefore, recommends that "When market milk is pasteurized it should be heated to about 145° Fahr. and held at that temperature for thirty minutes."

---

Transmutation of Elements. This expression, the hope of the ancient chemists, the object of scorn of modern ones, has, to some extent, been justified by recent studies of radiant energy. Collie and Paterson have shown that the bombardment of hydrogen by cathode rays produced helion, until recently regarded as a non-terrestrial element though common in the spectra of the sun and other stars. Apparently helion is a polymerized hydrogen, four atoms of hydrogen forming one of helion. Ramsay has continued the process, combining helion and oxygen to form neon; hydrogen and sulphur to form argon and hydrogen and selenium to form krypton. As has been alluded to previously, copper has been "degraded" into lithium. However, it is only fair to consider that most of these experiments, especially the majority which deal with radium and allied radio-active substances, apply the term *element* to rare and relatively unfamiliar substances. While it is entirely possible that the term *element* may come to have a significance quite different from its etymology and its present—or very recent—conception, and that we shall come to regard our whole present list of elements or most of them as merely polymeric forms of one or a few real elements or as compounds highly resistant to ordinary methods of disintegration, it is also quite as possible that both the conception of the word *element* and the essential elementary nature of most of the substances at present so considered will remain. In the latter case, we shall merely have to admit that chemists have been too hasty in giving elemental designations to a few unfamiliar substances.

---

MEDICAL SCHOOLS MERGED. The Atlanta College of Physicians and Surgeons and the Atlanta School of Medicine have been united under the title of the Atlanta College of Medicine, the original name of a school founded in 1854 and merged in 1888 with the Southern College to form the College of Physicians and Surgeons.

---

CONSOLIDATION OF MEDICAL SCHOOLS. Williamette University and the University of Oregon have merged their medical departments.

---

MEDICAL CLINICAL COURSE AT THE HOTEL DIEU, PARIS. Prof. A. Gilbert, under the auspices of the University of Paris, announces a course of clinical practice and application of laboratory methods, Sept. 22—Oct. 7, at 10.30 A. M. and 3 P. M. Aplicants should inclose an order for 100 Francs (about $20.00) to M. Deval, Chef de Laboratoire, Hotel Dieu, Paris. In March a similar course will be given on recent ideas on maladies of the Liver, Pancreas and Spleen.

---

TEN POLICEWOMEN have been assigned to duty in Chicago.

---

60,000 HORSES were eaten in Paris in 1911. The ordinary retail price is 3½ cents a pound. The meat is somewhat dry and insipid but resembles beef. Smoked and shaved, it appears and tastes like a superior quality of dried beef. It contains slightly more glycogen than most fresh meats.

ISOLATION HOSPITAL DE LUXE. Chicago will shortly have a hospital in which each patient will have an individual room, separated by a glass wall from visitors.

---

THE OTHER SIDE OF THE MASHING EVIL. A man has recently been released after serving twenty of a thirty days' sentence for street "mashing." It was proved that he had been condemned on perjured testimony. The editor of the *Critic and Guide* comments that no one but a drunken man will persist in annoying a woman who objects to his attentions and that a decent woman will not seek the notoriety of suit on such occasion. The plain fact exists that the evil of mashing exists because there are plenty of women who will accept the acquaintance of a stranger, not to mention the street walkers who court them. Some years ago, two lads started out to make a record. In a perfectly respectable though not very high-class residence district, their advances were accepted by twenty-seven girls, none of whom appeared to be immoral in the ordinary sense. In Europe, one sees men make advances to women in the most matter-of-fact way and the women, if respectable, shake their head with equal calmness, and there the matter drops. In this country, the wave of interest in or rather of open discussion of sex topics, has unavoidably caused some hysteria and judges should not forget that the desire to be in the limelight, rather than injured purity, is apt to be the origin of charges of accosting. And it should not be forgotten that there are times and places and classes or localities in which local custom excuses or even demands greetings among strangers. The legal precedent that a man may never speak to a woman whom he does not know, and vice versa, without liability to arrest, would lead not only to inconvenience and opportunity for blackmail, but, worst of all, to a state of sexual self-consciousness, which is a possible predisposing cause of sexual immorality. It would be well to limit the criminal recognition of "mashing" to plain and flagrant cases.

---

A PREMARITAL HEALTH CERTIFICATE for males will be required in Oklahoma.

---

A SPECIAL ROOM AND OPERATING EQUIPMENT FOR THE TREATMENT OF DENTAL DISEASE IN THE TUBERCULOUS has been established through the generosity of the Coterie, womans' society of Buffalo, with the co-operation of the Dental Department of the University of Buffalo. It will be in charge of Wm. J. Roche, D. D. S.

### The Educational Report of the A. M. A.

For many years the *Journal of the A. M. A.* has published, during the month of August, an elaborate report on medical education. This year's report adds a new feature—a diagram showing the period of existence of every medical school in the country from 1765 to the present, so arranged as to indicate mergers. We note that one school represents six original institutions.

Number of Schools.—High water mark was reached in 1906, when there were 162 schools, 130 of them regular. The maximum number of homeopathic colleges was in 1900 and 1901—22. The maximum number of eclectic colleges was in 1901—10. The last of the "physio-medical" and "non-descript" colleges passed away in 1910. At present there are 91 regular colleges, 10 homeopathic and 5 eclectic, a total of 106. However, excluding colleges whose discontinuance is announced in the near future or already reported, and excluding schools, mostly if not entirely departments of universities which do not confer degrees, but merely give the first two years in connection with collegiate departments, the real total for economic purposes is only 95.

Classes of Schools.—Class A plus, entirely satisfactory, number 22 (with the above mentioned exclusions), Class A, satisfactory except in minor details, number 33. Class B, needing general improvement, number 18, and Class C, requiring complete reorganization, 22. Three of the last class, however, are not recognized by their own state board as fitting men for a license, and while the aggregate number of students at schools of the first three classes is very nearly proportionate to the number of these schools in the total, the Class C schools, which comprise 25 per cent. of the total, have only 10 per cent of the students. Four of the five eclectic schools are included in Class C. The early extinction of the Class C schools, except for a few which will undoubtedly improve, is to be expected. For practical purposes, disregarding schools numerically and otherwise weak, medical education is almost entirely confined to about 90 schools, including 10 homeopathic and 1 eclectic.

This corresponds quite well with the theoretic estimate which we proposed last year, that the minimum economic basis for a medical school should be a million population, served by a thousand physicians, of whom about 20 would die in a year and require replacement by the annual graduating class, while the population would increase by about 1.5 per cent. a year, requiring about fifteen additional graduates, making the annual graduation 35. With a few more mergers and extinctions and the constantly increasing population, a still larger average medical school is within sight, enabling a better economic management.

Educational Standards. Without going into details and ignoring minor qualifications, it may be said that the four-year course in medicine, with terms of satisfactory length, and a minimum matriculation requirement of a high school education, has already been achieved. Six states already enforce a matriculation standard of two years of college study, five of one year. Each of these groups will be increased by one next year. Thirty-two medical schools already require two years of college study for matriculants, twenty-one require one year. Twenty-one more will require one year, beginning with the session of 1914-5 and seven will require two years in 1914-5 or 1915-6. These groups are duplicated to some extent, but it is a safe estimate that, within two years, there will not be more than fifteen schools in the country at which a man can matriculate without at least one year's college study beyond the high school course. A trifle less than 20 per cent. of this year's graduates in medicine hold bachelor's degrees.

Number of Graduates, Students, etc.—High water mark was reached in 1904, with 28,142 medical students and 5,747 graduates. There has been a fairly steady subsidence of this flood to the minimum of this year (since 1880-90) 17,015 students and 3,981 graduates. Last year the respective figures (previous low mark for students but not for graduates) were 18,412 and 4,483. Unless there has been a very uneven fluctuation in matriculation, which seems unlikely, it is probable that next year will show still better figures, for, while the number of graduates this year was 502 less than in 1912, the number of men left in the upper three classes, after Commencement last year, was 13,929, while this year it is only 13,034. Even if the rapid elevation of matriculation standards has a reactive influence in hurrying men into medicine, instead of a direct depressing effect, there can be no significant increase, but probably will be a decrease, for at least four years more, during which the weeding out process will have time to exert an influence.

Relation to Practicing Physicians.—There is presented a table of physicians and population, similar to that which we published in January, 1913, except that the A. M. A. Directory gives 142,190 physicians, whereas, our table, based on Polk's Directory, gives only 129,002. The Business Address Co. of New York at present carries a list of 139,585. The ratio of physicians to population varies considerably in different states, with an average of 1:713 or 1:640, according to the tables followed. We are inclined to believe that the lowest of these lists is about 10 per cent. high, if only physicians actually in practice to any economically appreciable degree are considered. To be conservative, it may be held that there are 120,000 physicians to a present population of about 97,000,000, giving an approximate average clientele of 1:800. This is much more favorable than has been supposed. It is an obvious paradox that the optimist in regard to the amelioration of an over-crowded profession should calculate mortality by the pessimist's statistics of practitioners, but it is fairer to base it on the low figures, since retired and non-practicing physicians are already removed from competition. In a profession of the age limits of physicians, the average mortality is somewhere about 2 per cent. per annum (about 13:1000 for the ages 25-34; 19:1000 for the ages 35-44; 38:1000 for the ages 45-64, and thereafter increasing very rapidly). Thus, to replace the deaths in the profession, about 2400 graduates would be needed annually. Meantime, the population is increasing at the rate of about 1,800,000 a year (a little less than 2 per cent. a year). On the same average basis of 800 clientele, this would accommodate about 2250 more graduates, making a total of 4650. This year there has been an actual shortage of 669 from this number. To put the matter in a different way, a maintenance of the present ratio of physicians to population would be represented by an annual graduation of about 4 per cent. of the number of the practicing profession, 2 per cent. to compensate for mortality, 2 per cent. to correspond to increase of population, while at present the number of graduates is about 3 1/3 per cent. This means a very slight amelioration of existing economic conditions—an average addition to the clientele of about one family of five persons a year. But, the tide seems to have turned.

We cannot close this article without calling attention to the enormous amount of labor represented in the compilation of these statistics by the A. M. A. and their great value. But the statistics themselves are insignificant compared with the work for the advancement of the profession, and thus, for humanity, done by the A. M. A., and by the forces which it has supported and to a large degree instituted. In less than a decade the standards of medical education have been advanced far beyond what was hoped for as a realizable reform, almost indeed to the point regarded as the ideal of idealists. We have dwelt largely on economic considerations, but these are of minor consequence in comparison with the ethical, educational and ultimately humanitarian improvements which have ceased to be dreams and have become realities. Let each reader ask himself whether he is doing his duty in supporting professional organization and in lending his influence to the attainment of ideals in that organization.

---

### Laws of New York, Chapter 526

An Act to Amend the Insanity Law by authorizing any licensed private institution for the insane to receive inebriates for commitment and care. Became a law May 15, 1913, with the approval of the Governor. Passed, three-fifths being present. The People of the State of New York, represented in Senate and Assembly, do enact as follows:

Section 1.—Chapter thirty-two of the laws of nineteen hundred and nine, entitled "An act in relation to the insane, constituting chapter twenty-seven of the consolidated laws," is hereby amended by adding at the end thereof four sections, which shall be known as sections one hundred and seventy-three, one hundred and seventy-four, one hundred and seventy-five and one hundred and seventy-six, to read as follows:

Sec. 173.—The judge of a court of record in the county or district where an alleged inebriate resides, or a judge of any court of record, may commit such person to any private licensed institution for the insane, in the manner hereinafter provided, upon a proper application and upon the consent in writing of the trustees, signed by their superintendent or executive officer, upon the certificates in writing made, executed and verified by at least two physicians, qualified to act as medical examiners in lunacy, showing that such person is over the age of eighteen years, and is incapable or unfit to properly conduct himself or herself, or his or her affairs, or is dangerous to himself or herself or others by reason of periodical, frequent or constant drunkenness, induced either by the use of alcoholic or other liquors, or of opium, morphine, or other narcotic or intoxicating or stupefying substances. Such certificate must further show that such person is in actual need of special care and treatment, and that his condition is such that his detention, care and treatment in such institution would be likely to effect a cure. Such certificate shall also specifically state the facts and circumstances upon which the judgment of each physician is based and shall show the result of such examination. It must appear upon the face of such certificate that each physician executing the same has made a personal examination of the person alleged to be an inebriate, and that such an examination has been made within ten days prior to the application for the commitment.

Sec. 174.—The husband or wife, father or mother, brother or sister, or the child or committee of an alleged inebriate may apply for an order committing such person to the said licensed private institution for the insane, by presenting a brief petition containing a statement of the facts because of which the application for the order is made. Such petition shall be accompanied by the certificate of the physicians and the consent of the trustees as prescribed in the preceding section. Notice of the time and place of making such application shall be served personally upon the alleged inebriate at least three days before the date therein specified upon which the application will be made. A copy of the petition shall be served with such notice. The judge or justice before whom such application is made shall, in his discretion, direct the service personally or by mail of a like notice upon the husband or wife, father or mother, or next of kin, of such alleged inebriate. At the time and place mentioned in such notice or at such other time or place as the judge or justice may designate, said judge or justice shall proceed to hear the testimony introduced for and against such application, and may examine the alleged inebriate if deemed advisable. Such judge or justice may, in his discretion, require proofs in addition to the petition and certificates of the physicians. If, from the facts ascertained upon the hearing, the proofs produced, and the petition and certificates presented, the judge or justice shall determine that such person is an inebriate, or that he is so addicted to the use of opium, morphine or other narcotic or intoxicating or stupefying substance, and his condition is such that his detention in such institution would promote his interests and improve his health, he shall grant an order committing such person to such institution, to be detained therein for a period not exceeding twelve months, or for such period less than twelve months as may be necessary in the judgment of the physician in charge of such institution for the proper treatment and cure of such person, or until discharged therefrom prior to the expiration of such period, as hereinafter provided. The physician in charge may grant a parole to a patient not exceeding six months.

Sec. 175.—A person committed pursuant to this act or any relative or friend in his or her behalf may, within thirty days after any order of commitment is granted as provided in the preceding section, apply to a justice of the supreme court other than the justice making the commitment for a review of such order. Such justice shall thereupon cause a jury to be summoned as in the case of the proceedings for the appointment of the committee for an insane person, and shall try the question of the inebriety of such person in the manner provided by law for the proceedings for the appointment of such committee. If the verdict of the jury be that such person is an inebriate, such justice of the supreme court to whom such application was made shall certify that fact and commit such person to the care and custody of the said institution. Proceedings under the commitment shall not be stayed pending an appeal therefrom, except upon an order of a justice of the supreme court made upon notice and after a hearing, containing a provision for such temporary care or confinement of the alleged inebriate as may be deemed necessary. Upon the refusal of a judge to grant an application for the commitment of an alleged inebriate he shall state his reasons for such refusal in writing, and the person making the application may apply to a justice of the supreme court in the manner specified in this section where an application is made in behalf of the alleged inebriate, and a commitment may be had after an appeal by a jury as provided herein.

Sec. 176.—A person who has been committed to such institution is entitled to a writ of habeas corpus upon a proper application made by him or her or by any relative or friend in his or her behalf; upon the return of such writ, the fact of the inebriety of such person and the reasons for his or her further detention in such institution shall be inquired into. The superintendent or executive, or the medical officer in charge of such institution, or any proper person, may be sworn and examined, as to the mental and physical condition of such person. If it appears upon such hearing that such person may properly be discharged, the judge or justice before whom the hearing is had shall so direct; but if it shall appear that the condition of such person is such as to render further treatment desirable, such person shall be remanded to the care and custody of such institution.

Sec. 2.—This act shall take effect immediately.

---

Water and Ice Supplied by Interstate Carriers. On January 25, 1913, the Secretary of the Treasury, under authority of an act of Congress approved February 15, 1893, promulgated the following regulation regarding the water and ice furnished to passengers by common carriers in interstate traffic:

---

Amendment to Interstate Quarantine Regulations.

Article 3, General Regulations, is hereby amended by the addition of the following paragraph:

"Paragraph 15. Water provided by common carriers on cars, vessels, or vehicles operated in interstate traffic for the use of passengers shall be furnished under the following conditions:

"(*a*) Water shall be certified by the State or municipal health authority within whose jurisdiction it is obtained as incapable of conveying disease: *Provided,* That water in regard to the safety of which a reasonable doubt exists may be used if the same has been treated in such manner as to render it incapable of conveying disease, and the fact of such treatment is certified by the aforesaid health officer.

"(*b*) Ice used for cooling such water shall be from a source the safety of which is certified by the State or municipal health authority within whose jurisdiction it is obtained, and before the ice is placed in the water it shall be first carefully washed with water of known safety and handled in such manner as to prevent its becoming contaminated by the organisms of infectious or contagious disease: *Provided,* That the foregoing shall not apply to ice which does not come in contact with the water which is to be cooled.

"(*c*) Water containers shall be cleansed and thoroughly scalded with live steam at least once in each week that they are in operation."

---

### Instructions Relative to the Certification of the Water and Ice Furnished to Passengers in Interstate Traffic.

Samples of water and artificial ice from each and every source of supply should be subjected to bacteriological and chemical examination at least once in every six months by the proper State or municipal health authority within whose jurisdiction the supply is obtained, or by other person or persons competent to make such examinations and whose results will be accepted by the State or municipal health authority whose duty it is to issue certificates. Each new crop of natural ice should be examined and certified before use.

The common carrier desiring a certificate of the State or municipal health authority within whose jurisdiction the water or ice is obtained should make application therefor.

After the necessary examinations shall have been made the certificate should be issued on the form supplied, one copy to be delivered to the common carrier, one copy to be forwarded to the Surgeon General, United States Public Health Service, Washington, D. C., and one copy to be retained as a matter of record and for future reference.

Whenever there is an unusual prevalence of typhoid fever, dysentery, infantile diarrhoea, or other water-borne disease in a locality from which common carriers receive water and ice, an additional examination of the water and ice should be made and a supplemental certificate made by the proper certifying authority and forwarded as above.

---

Damage Suit by Sterilized Patient. Two years ago an inmate of the Outagamie Co. Asylum for the Insane, Wisconsin, was sterilized. He has regained his sanity and now, through his guardian, is suing the superintendent and the former county physician on account of the operation.

U. S. Civil Service Examination, Professor of Pharmacology. There is at present a vacancy in the Hygienic Laboratory, U. S. Public Health Service, Washington, at a salary of $4,500, and similar positions may later be available. No formal examination will be held, but selection will be made according to evidence of fitness presented by candidates, who should apply immediately for Form 1312, to the U. S. Civil Service Commission, Washington, as the form must be properly executed and filed before September 15. Applicants must be under 45, must have a Ph. D. degree or show equivalent study, and must have devoted at least ten years to pharmacology and closely allied branches.. Special credit will be given for original publications of merit.

---

Lay Control of Operations. The Olean Common Council on August 18, failed to pass an ordinance providing for a board of physicians of twenty years' experience, who should examine all patients on whom it was proposed to perform an operation. The committee to whom the proposed ordinance had been referred made a somewhat sensational report, charging that many operations were performed for financial reasons. We are glad that the majority of the Council were sensible enough to vote against such a measure. It is very questionable whether such a local ordinance would be sustained by the courts and obvious that if sustained, it would lead to many vexations and possibly to opportunities for venting professional hostility and for black mail. For instance, it is difficult to define the word operation in accordance with the probable intent of the proposed ordinance. Imagine a patient with an aching and hopelessly decayed tooth waiting for the examination of the committee of physicians of twenty years' experience—not in dentistry—or a similar delay before lancing a boil. But, where there is much smoke, there is some fire. Similar complaints and suggestions crop out in various places, from time to time. So far as we have personally observed, the furor operandi is due rather to ingrowing professional ambition and unilateral skill than to mercenary motives, but the results are equally bad. And the whole problem is so easily solved! All that is necessary is a reasonable disposition to share grave responsibilities and to acknowledge the value of the opinions of others than one's self. Imagine a competent, experienced physician, in the limited sense, essaying a difficult operation. Unless the circumstances were such as to preclude the possibility of securing expert surgical aid within the necessary time such a man would be universally condemned. Excepting acute, uncomplicated cases of traumatism—granting that they really are uncomplicated—almost every major operation invokes problems in visceral physiology, nutrition, elimination, etc., which deserve just as skillful attention as the purely surgical details. And, in many other instances, there is room for a broad consideration of the best means of therapy. We have personally experienced the harrowing sight of an inexperienced operator slashing into the abdomen, losing his nerve and falling back on the interne and an equally inexpert medical man. And the memory of that experience is not softened by the necropsy which showed no apparent indication for operating at all. We have, in anticipation, felt what it must mean to operate, realizing one's incompetence, but realizing also that the absolute impossibility of obtaining surgical aid necessitated giving the patient the only chance. But the memory of this experience is assuaged by the fact that the dilemma was solved by the favorable course of the case without intervention. If all operators could realize that the neglect of matters which they consider of minor importance or which they do not realize at all, is the exact counterpart of a major operation performed by one who has no surgical skill, there would be no excuse for freak legislation to control operations by license.

---

THE BINGHAMTON STATE HOSPITAL graduated eleven nurses in July.

---

THE VANDERBILT UNIVERSITY MEDICAL COLLEGE, in spite of rumors to the contrary, has decided that Andrew Carnegie's endowment of a million dollars is sufficiently asceptic to accept. This is a proper action. In fact, we would almost go so far as to say that the only money solicited for education and benevolence on a large scale should be that raised by equable taxation and what has been called "tainted."

The Association of Medical Instruction of the Hospitals of Paris will give a series of conferences at the Charity Hospital, October 15-31, three hourly lectures being given each afternoon, beginning at 3.30. The topics and the lecturers will represent various branches of medicine.

